# An engineering model of the COVID-19 trajectory to predict the success of isolation initiatives

**DOI:** 10.14324/111.444/ucloe.000020

**Published:** 2021-06-30

**Authors:** Steven King, Alberto Striolo

**Affiliations:** 1 Department of Chemical Engineering, University College London, Torrington Place, London WC1E 7JE, UK

**Keywords:** virus propagation model, engineering approximations, length of intervention

## Abstract

Much media and societal attention is today focused on how to best control the spread of coronavirus (COVID-19). Every day brings us new data, and policy makers are implementing different strategies in different countries to manage the impact of COVID-19. To respond to the first ‘wave’ of infection, several countries, including the UK, opted for isolation/lockdown initiatives, with different degrees of rigour. Data showed that these initiatives have yielded the expected results in terms of containing the rapid trajectory of the virus. When this article was first prepared (April 2020), the affected societies were wondering when the isolation/lockdown initiatives should be lifted. While detailed epidemiological, economic as well as social studies would be required to answer this question completely, here we employ a simple engineering model. Albeit simple, the model is capable of reproducing the main features of the data reported in the literature concerning the COVID-19 trajectory in different countries, including the increase in cases in countries following the initially successful isolation/lockdown initiatives. Keeping in mind the simplicity of the model, we attempt to draw some conclusions, which seem to suggest that a decrease in the number of infected individuals after the initiation of isolation/lockdown initiatives does not necessarily guarantee that the virus trajectory is under control. Within the limit of this model, it would seem that rigid isolation/lockdown initiatives for the medium term would lead to achieving the desired control over the spread of the virus. This observation seems consistent with the 2020 summer months, during which the COVID-19 trajectory seemed to be almost under control across most European countries. Consistent with the results from our simple model, winter 2020 data show that the virus trajectory was again on the rise. Because the optimal solution will achieve control over the spread of the virus while minimising negative societal impacts due to isolation/lockdown, which include but are not limited to economic and mental health aspects, the engineering model presented here is not sufficient to provide the desired answer. However, the model seems to suggest that to keep the COVID-19 trajectory under control, a series of short-to-medium term isolation measures should be put in place until one or more of the following scenarios is achieved: a cure has been developed and has become accessible to the population at large; a vaccine has been developed, tested and distributed to large portions of the population; a sufficiently large portion of the population has developed resistance to the COVID-19 virus; or the virus itself has become less aggressive. It is somewhat remarkable that an engineering model, despite all its approximations, provides suggestions consistent with advanced epidemiological models developed by several experts in the field. The model proposed here is however not expected to be able to capture the emergence of variants of the virus, which seem to be responsible for significant outbreaks, notably in India, in the spring of 2021, it cannot describe the effectiveness of vaccine strategies, as it does not differentiate among different age groups within the population, nor does it allow us to consider the duration of the immunity achieved after infection or vaccination.

## Introduction

The development of the coronavirus (COVID-19) pandemic both in terms of its geographical footprint and the growth of cases and fatalities has been the subject of opportune comment and provided the news media with a constant and compelling feed since the end of 2019. Along with several detailed analyses of the spread of the pandemic in different parts of the world [1–3], current studies address the impact of easing the restrictions imposed to contain the spread of the virus [4], descriptors that enhance or curtail the negative impact of an infection on humans [5,6], the development of animal models to eventually test a vaccine [7], the physiology of the virus itself [8–10], ethical aspects related to the development of a vaccine [11–13], plans for the distribution of vaccines [14] once they are developed, as well as the impact of different mitigation strategies on the viral trajectory [15]. This list is not exhaustive, given the tremendous importance of the topic. Concerning predictions regarding the spread of the pandemic, wide variations are noted on the expected future outcomes, both at the time of writing and when the first version of this article was prepared (in April 2020). To overcome these wide variations, modelling has been attempted here using an engineering differential model, which, if successful, could provide an evidence-based prediction of future expectations once some parameters are fitted to the hard data. It should be emphasised that the model we seek to develop has many simplifications, and its main attributes are the ease of use and the ability to reproduce available datasets. Therefore, an SIR-type model was chosen because it has a long history in modelling infection propagations in populations [16]. SIR models consider three classes of individuals: S – susceptible; I – infected and R – recovered (or deceased). The approach was developed in 1927 by Kermack and McKendrick [17], and indeed it has been successfully applied since to the modelling of large historical epidemics. Both the capacity and limitations of SIR type models are well understood and documented [18]; for example, their assumptions somewhat limit their ability to completely describe infection numbers, and the connection between infected period and potential to spread infection is not included as a critical parameter [19]. The SIR model formulation has been extended to acknowledge the influence of other parameters [1,20]; the effects of population mixing and variations in size have been examined [21] and it has been acknowledged that minor changes in the model parameterisation can produce predictions of complicated behaviours. Including these additional parameters would yield models capable of describing complicated infection dynamics to effectively model the trajectory of the COVID-19 infection but would require knowledge of a greater number of parameters. Therefore, we made the conscious choice of applying the basic formulation of the SIR model in our analysis.

The purpose of this study was to model the trajectory of the COVID-19 infection with specific consideration in modelling the effect of isolation/lockdown directives on said trajectory. Before the advent of effective vaccines, it became apparent that the degree of compliance to isolation/lockdown directives is the most effective strategy to manage the physical footprint of the virus [22,23], which seems to validate the conclusions achieved by the implementation of simple SIR models.

A simple model such as the one chosen here offers the advantage of easily identifying the defining equations and the governing parameters, with the benefit of explicit coupling of isolation/lockdown effectiveness to rate constants. Then the quest was whether such a minimal model was still capable of providing useful predictions on future possible trajectories of COVID-19 infections. As our model was constructed during the early period of the COVID-19 pandemic, when response strategies such as social controls, dedicated hospitals and travel restrictions were being introduced, the most robust modelling strategy was considered to be the one involving the least number of unknown parameters. Although we recognise that more complete models provide a physical description of the mechanisms of spreading and recovering from the infection, among others, the merits of simple models in terms of ease of understanding of the implications of directives and public behaviour have been widely acknowledged [24,25]. Since the first draft of this article was produced (April 2020), the rapid development of the pandemic in terms of physical spread and local case numbers has provided additional data, which enabled further testing of the reliability of the engineering model proposed in the first revision of the article (October 2020). Certainly, such an exercise could be repeated multiple times as new data becomes available from different locations around the world, but this is not the goal of our approach. Since the first revision of the article in October 2020, the introduction of vaccines has contributed to bringing the trajectory of the COVID-19 virus under control [26–29] but the emergence of new variants and mutations [30–32], most notably in India but also elsewhere, reminds us of the possible challenges for vaccines and immunotherapies alike.

COVID-19 trajectory data, used to derive and to validate the model, was sourced from the World Health Organization (WHO), via https://ourworldindata.org/coronavirus-source-data [33], which provides current daily new case and mortality figures for most countries. Data as of 2 April 2020 were used for the analysis presented in the first version of this article, with a review of trends being undertaken on 21 September 2020. It should be noted that the data chosen for our analysis reflect situations with relatively high population density.

## Methodology

The differential model employed was constructed as follows, based on a population of fixed size (Po), in which three groups of Individuals were defined:

1. X = uninfected Individuals;
2. Y = infected Individuals;
3. REC = Individuals not able to pass on infection by virtue of recovery, or fatality.

Defining

k_1_ = infection growth rate constant, which will itself be a function of the frequency of daily person-to-person contact (assumed random) and of a yet unknown efficacy of transfer;

and

k_2_ = the rate constant for the recovery/mortality of the infected population (Y).

The following 1^st^ order differential equations may be defined:

[1] $$\frac{\text{dX(t)}}{\text{dt}}{\text{=−k}}_{\text{1}}\text{X(t)Y(t)}$$

[2] $$\frac{\text{dY(t)}}{\text{dt}}{\text{=k}}_{\text{1}}{\text{X(t)Y(t)−k}}_{\text{2}}\text{Y(t)}$$

[3] REC(t)=Po−X(t)−Y(t)

The above non-linear equations [1–3] may be solved numerically, for example, using a Runge–Kutta–Simpson technique [34], with the initial conditions being X (0) = Po, Y (0) = 0, REC (0) = 0.

It should be emphasised this model only partitions the population into three groups, with no spatial differentiation, nor distinction among the age groups; the geographical position of population elements is not considered, and the population is considered fixed. This is consistent with the spirit on an engineering model, in which the combined effects of these differences would result in different values for the few parameters used to apply the model to a given case study (i.e., fitting parameters).

It could be useful to provide some semi-quantitative guidance to relate the parameters in Eqs. [1–3] to those of some advanced models recently reported in the literature [16]. Explicitly, in our model the parameter k_1_ is the infection growth rate constant, also indicated as intrinsic growth rate, whose units are persons^–1^ time^–1^; this parameter may be considered as a *per capita effective contact rate*. Hethcote uses the parameter β to represent the contact rate, expressed in the units of (time^–1^) [16]. We note that the following relation relates k_1_ and β as:

[4] $${\text{β=k}}_{\text{1}}\text{N}$$

In Eq. [4], N represents the total population (N = X + Y + REC = Po), which is considered fixed in our model.

Further, in our model, the parameter k_2_ is the rate constant for recovery/mortality (time^–1^); to connect with the approach described by Hethcote, we note that k_2_ is related to the average infection time, sometimes indicated by λ (expressed in the units of time), via the relation:

[5] $${\text{k}}_{\text{2}}\text{=1/}\lambda$$

In the statistical models used to describe the COVID-19 trajectory, extensive reference has been made to the basic reproduction number R_0_, which, in the review of mathematical modelling [19], is defined as the average number of infections passed on by an infected individual; it is a dimensionless ratio and is related to the parameters discussed in our model (Eqs. [1–3]), via:

[6] $${\text{R}}_{0}{\text{=βλ=k}}_{\text{1}}{\text{N/k}}_{\text{2}}$$

The relations shown by Eqs. [4–6] provide a key to translating the results obtained by the engineering model developed here in terms of data presented in the literature by other modelling approaches. This correspondence suggests that, even though the model developed here is minimalistic, it could be compared against the predictions obtained using more complicated approaches.

**Figure 1:**
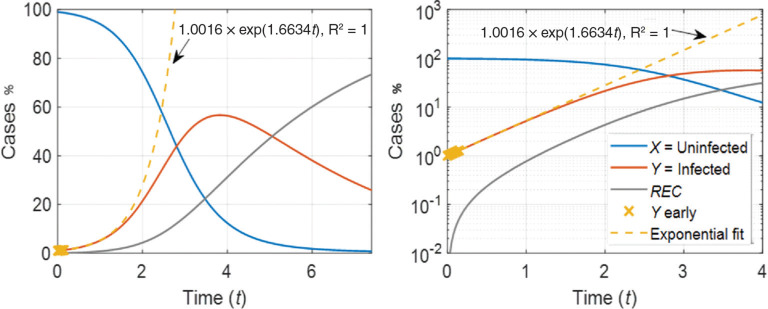
Differential model to describe the propagation of a virus through a total population (Po) which is initially completed uninfected (X, blue line), and, as time progresses, becomes infected (Y, orange line) and then either recovers or dies (REC, grey line). The yellow dashed line is an exponential growth model fitted to the early growth stages (yellow symbols) of the infected population Y. In the left panel, the model is presented in linear representation, while in the right panel the logarithmic representation is used. The vertical axes ‘cases’ represent percentage values. Parameters values are k_1_ = 0.02, k_2_ = 0.3, and P_0_ = 1%.

The development of the three groups of Individuals (X, Y and REC) with time as predicted by our model is shown in Fig. 1 in both linear and logarithmic scaling representations. In Fig. 1, the dotted line indicates an exponential growth of the infected population, which is fitted to the early part of the correspondent curve [i.e., Y(t)]. Although a very basic model. The character of the curves is consistent with actual infection transfer rates as well as with other models presented in the literature [1,19,32]. Fitting is not shown here because abundant analysis is reported on the news as well as on specialist literature [2,16–18]. The most significant feature evident from our engineering representation of the infected population Y curve is its eventual departure from exponential growth, evidenced by the change in colour in the Y(t) curve in Fig. 1, as the trajectory of the disease continues. This departure evidences the possibility of reaching ‘the peak’ in the infection trajectory and eventually reaching ‘herd immunity’. We acknowledge that the acceptance of herd immunity as an outcome of a viral trajectory requires that the majority of the population (perhaps 80% as suggested by some analysis) experiences infection and those that survive and secure immunity act to terminate future infective growth chains. This ‘do nothing’ approach ultimately seeks to allow the infection trajectory to take its course, but it can come at enormous cost, potentially borne inequitably on those most susceptible. It should however be recognised that in some communities (notably in Sweden), a deliberate choice was made to not impose restrictions on individual freedoms, potentially with the goal of achieving such ‘herd immunity’. Time will tell which approach has been able to curtail the pandemic without allowing for too much unintentional negative impact to be delivered.

In the engineering model presented here, the departure from exponential growth in the number of infected Individuals is due to larger number of contacts between infected Individuals as opposed to contacts between infected and uninfected ones, recovery or death of the infected Individuals (described by the constant k_2_), and reduced total population, all eventualities which effectively would eventually terminate the growth chain.

Analysing the results shown in Fig. 1, it may be seen that prior to Y reaching 2% of Po (at time = 0.4157) the correspondence to exponential growth approximation is very good, with Y model/Y exponential = 1.0009. Given this good agreement, it is possible to render the model a-dimensional by expressing the Y(t) curve as a deviation from the correspondent exponential growth model Y_exponential_(t), under the constraint of limiting the analysis to Y < 2% of Po. Using the a-dimensional model it is then possible to draw generally applicable conclusions independently on the actual values of Po, k_1_ and k_2_. The time units have not been defined in this case, to emphasise that the analysis of the system behaviour is independent of the time unit and rate constant units.

The significance of the above observation is that in examining WHO data, where the infected population (Y) is a very small proportion of the total (or local) population Po, for unchanging rate constants k_1_ and k_2_ a simple exponential growth in Y should be observed. From our engineering model:

[7] $$\frac{\text{dY(t)}}{\text{dt}}{\text{=k}}_{\text{1}}{\text{X(t)Y(t)−k}}_{\text{2}}\text{Y(t)}$$

Because X ∼ Po in our approximation of Y < 2% of Po,

[8] $${\text{k}}_{\text{3,initial}}{\text{=k}}_{\text{1}}\text{Po}$$

[9] $$\frac{\text{dY(t)}}{\text{dt}}{\text{=(k}}_{\text{3,initial}}{\text{−k}}_{\text{2}}\text{)Y(t)}$$

Eq. [9] can be solved to yield

[10] $${\text{Y(t)=exp((k}}_{\text{3,initial}}{\text{−k}}_{\text{2}}\text{)t)}$$

It is in fact possible to apply Eq. [10] to WHO data, specifically, for different countries and regions, until the onset of isolation/lockdown initiatives, which have the goal of slowing down, and eventually reversing the growth rate. In other words, Eq. [10] represents an un-moderated exponential growth in the number of infected Individuals.

## Modelling the effect of isolation/lockdown initiatives

One timely question in relation to governmental initiatives designed to mitigate the spread of a virus, is quantifying the merits (and consequences) of strong and weak compliance to governmental health directives (i.e., social isolation and lockdown initiatives).

The effect of these initiatives with respect to the above engineering model is expected to introduce a stepwise down shift in the exponential growth constant for the disease, that is, k_3_ in Eq. [10]. This effect was modelled here by applying various stepdown factors (K_step-down_) to the growth constant k_3_ at the time when 1% of the total population, Po, was infected. In other words, at t > t _intervention_, we set:

[11] $${\text{k}}_{\text{3,intervention}}{\text{=k}}_{\text{3,initial}}\times (1-{\text{K}}_{\text{step-down}})$$

Note that K_step-down_ is limited between 0, which reflects an ineffective imposition of governmental initiatives, and 1, in which case said initiatives are so effective that no new Individual is infected. In our approach, the rate of recovery/mortality from the disease, k_2_, is considered unchanged.

**Figure 2:**
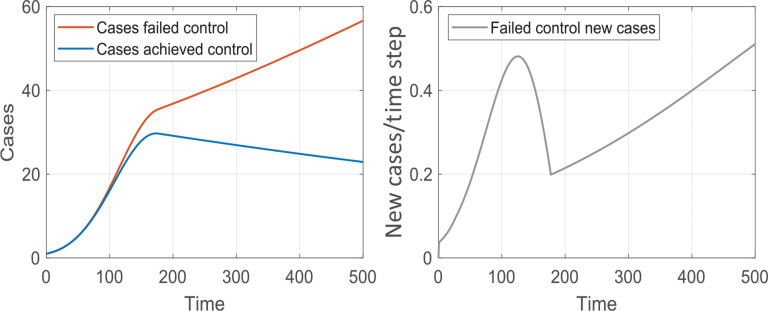
Left panel: Changes in the number of infected Individuals after isolation/lockdown initiatives are introduced. Two types of behaviour are observed, depending on the level of populace compliance with the guidelines. When compliance is high, the infected population decreases (blue curve); when compliance is not sufficiently high (orange), the exponential growth in the number of infected Individuals continues at a reduced growth rate. Right panel: The number of new infected Individuals is plotted as a function of time for the case shown in orange in the top panel. Cases as percentage figures. Time units are not defined.

Modelling Y(t) under various scenarios for a given k_3_ yields evidence that two behaviours emerge, depending on whether K_step-down_ is larger or smaller than a critical value. In the former case, the number of infected Individuals declines and over time control over disease prevails. When K_step-down_ is lower than the critical value, which corresponds to a lower degree of populace compliance with governmental health initiatives or ineffective governmental initiatives, Y(t) resumes its exponential growth and control over disease propagation is lost. In Fig. 2, the two behaviours are shown; control is achieved (blue) for K_step-down_ = 0.94, and not achieved (orange) for K_step-down_ = 0.90. This analysis yields K_step-down,critical_ = 0.92. It is instructive to analyse the number of new infected cases per day as predicted by our model when K_step-down_ = 0.90. These results are shown in the right panel of Fig. 2. It can be seen that while the number of new infected Individuals as a function of time (e.g., per day) initially decreases, an insufficient compliance with the governmental directives (or ineffective governmental initiatives – note that our model cannot distinguish between the two scenarios) eventually leads to growth in the number of newly infected Individuals.

For the engineering model to be helpful, one might ask how it is possible to determine K_step-down,critical_ depending on the initial population size, Po, and the infection growth rate, k_1_. As shown in Eq. [8], k_3_ is the product of these two values. Modelling reveals a simple relation between K_step-down,critical_ and k_3_:

[12] $$(1-{\text{K}}_{\text{step-down,critical}})\times {\text{k}}_{\text{3}}=\text{constant}$$

The relation represented by Eq. [12] holds independently of the point in time at which the isolation/lockdown initiatives are applied, although for the purposes of modelling, it is considered that the initiatives are applied during the early unmoderated exponential growth part of the curve, before a significant proportion of the population is infected (i.e., Y < 10% Po). It can be seen from Eq. [12] that the larger k_3_ is, the larger K_step-down,critical_ must be to achieve control over the spread of the virus. The engineering model predicts that the longer it is waited to impose isolation/lockdown initiatives after the initial appearance of the virus, the higher must K_step-down,critical_ be to achieve the desired effects. A longer delay in implementing isolation/lockdown strategies will also increase the number of infected Individuals.

The consequences of applying different K_step-down_ values may be modelled to assess the time frame of recovery. Such a time frame can be quantified by the correspondent recovery rate constant extracted from fitting to the exponential decay functions the blue portion of curves such as those in Fig. 2. Sample results are tabulated in Table 1, in which only K_step-down_ values above K_step-down,critical_ were considered. As smaller recovery constants are consistent with a very much slower rate of decline in the population of infected Individuals, thus defining a longer period of imposed intervention, this simple analysis clearly suggests that minimum discomfort, including economic cost, is achieved by application of the most severe intervention possible, to recover control in the shortest time frame.

**Table 1.** Recovery rate constants obtained by fitting the decay in the number of infected Individuals (e.g., Fig. 2) with an exponential function

k_step-down_	k_recovery_
0.990	1.715
0.985	1.574
0.980	1.434
0.975	1.296
0.970	1.159
0.965	1.025
0.960	0.889

The rate constants change as the K_step-down_ value increases above a critical value, as shown in the datasets below.

To quantify whether the engineering model and its predictions are reliable, one should fit the decaying functions to available datasets.

## Examples of intervention: China, South Korea and Singapore

The WHO database was examined because it provides up-to-date time sequences of new infective cases, total infections and deaths, broken down into nations. The original choice of a model dealing with infection levels rather than mortality figures was deliberate as the infective levels model is much simpler than one attempting to predict disease outcomes given the acknowledged correlation of age on outcome and the additional effect of the quality of available health care.

To assess the reliability of the engineering model presented here, China, South Korea and Singapore were chosen as examples of intervention based on several criteria, including:

1. Early encounter with disease; proximity to the origin of the disease meant that all three nations experienced growth in effective numbers early, with the result that the consequences of intervention were well defined. Many other nations which had a delayed encounter with the COVID-19 pandemic were experiencing unmoderated exponential growth at the time of writing, and thus provide no evidence to assessing the consequences of intervention.
2. Substantial cohort numbers; in association with point 1, the examination of large national cohorts of infection will act to reduce the noise in the time sequence and allow a better assessment of the correspondence between model and actual data.
3. Cultural similarity; the exponential growth constant defined for the disease is determined by the frequency and efficacy of transfer by immediate (person–person) or secondary (person–object/airborne–person) contact. It is appreciated that this is strongly influenced by cultural norms of social contact. Similarly, the effectiveness of disease control measures, such as social distancing, increased sanitisation and enforced lockdowns will be defined by the cultural norms of the societies concerned. As such, choosing three nations which are acknowledged as culturally similar provides a common basis to support valid comparison. The use of Hofstede’s index of cultural similarity is employed to this end, with the three nations concerned being defined as culturally similar [35].^1^
4. For the cases of South Korea and China [36–38] the number of new infected Individuals per day was plotted versus the total number of infected Individuals. The relationship is of the form:
[13] $${\text{Z(t)=Y(t)×(exp((k}}_{\text{3}}{\text{−k}}_{\text{2}}\text{)×Δt)−1}$$

In Eq. [13], Z(t) is the number of new infected Individuals per day, Y(t) is the total number of infected Individuals and Δt is the time interval between data points, usually a day. The data are presented over several orders of magnitude in a log–log plot, which shows the expected linear relationship typical of unmoderated exponential growth. Deviation from this linear relationship at low levels of infection is evidence of the control of the disease through effective governmental initiatives and public adherence to social distancing and lockdown guidelines, which have reduced the growth constant. In some cases, changes in behaviour can be the natural response to the perceived risk of infection, and not necessarily due to government intervention.

Comparison of the salient features of the two curves (unmoderated growth and moderated growth) may be achieved through quantification of two dimensionless ratios, which may be used to compare the two countries. The two ratios are A/B and C/D, where the letters have the following meanings:

- A represents the extrapolated number of cases per day which would have been expected from the exponential growth at the case number asymptote;
- B is the maximum encountered cases per day;
- C is the number of cases at the time of maximum number of new cases per day;
- D is the disease propagation asymptote, representing the total number of infected Individuals at the conclusion of the outbreak, when local control has been achieved.

**Figure 3:**
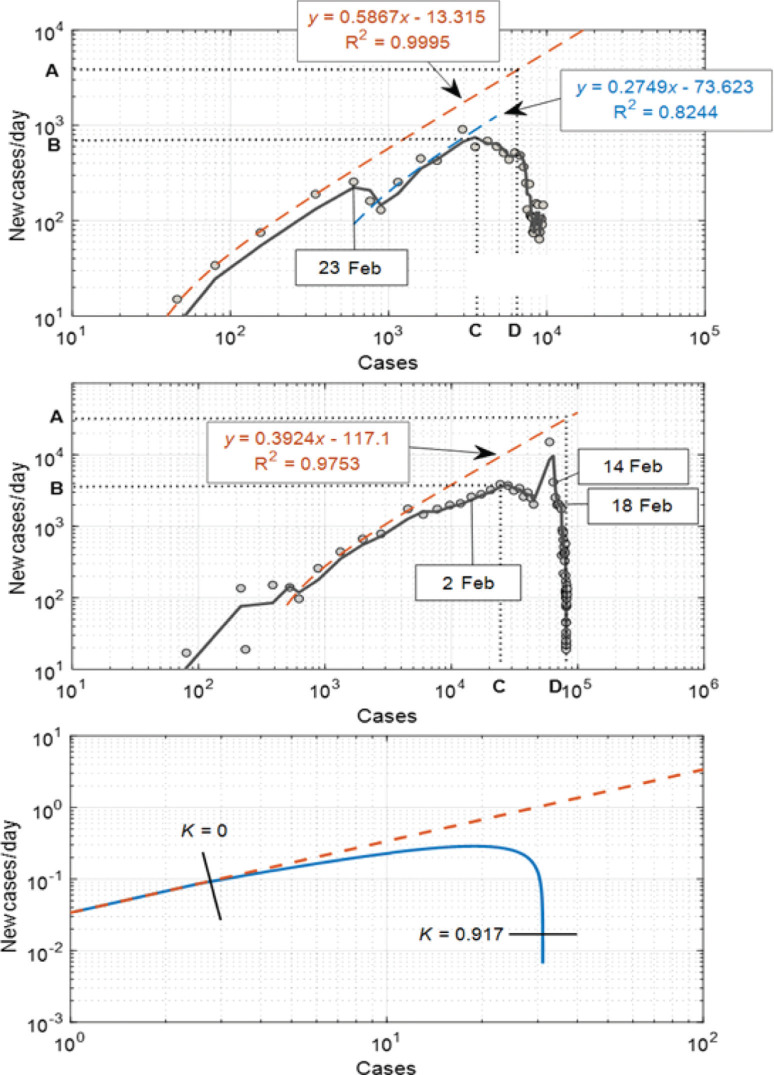
New cases/day versus total cases for South Korea with initial unmoderated exponential growth in orange. In blue we highlight the growth rate after the isolation/lockdown initiatives were implemented. In the top panel we report analysis for South Korea, with 23 February – the date of the first government self-isolation – isolated. Further initiatives were undertaken on 14 and 18 February, as indicated. In the middle panel we report the analysis of data from China. In the bottom panel we present model results in which K_step-down_ varies linearly over time, as described by Eq. [13]. The plot clearly shows that the number of infected Individuals per day decreases as K_step-down_ increases.

The data reported by WHO for South Korea and China, plotted in the formalism of Eq. [13], are plotted in Fig. 3. The points A, B, C and D are extracted from the graphs for these two case studies.

## Extension of the engineering model

In order to reproduce the curves in Fig. 3, a varying K_step-down_ parameter was applied, rather than a constant value as was the case in Eq. [11], via:

[14] $${\text{K}}_{\text{step-down}}=1-\text{const}\times {\text{t}}^{\text{n}}$$

Eq. [14] reflects the fact that compliance with social distancing and lockdown directives took time to take effect and thus K_step-down_ decreases with time to some final value. Fitting Eq. [14] to the data via the model detailed earlier yields the graphical results plotted in the bottom panel of Fig. 3.

The results show the progression of the new cases versus total cases for K_step-down_ transitioning linearly (n in Eq. [14] equals 1) with time from K = 1 down to K = 0.083. Only when K_step-down_ has reached values very close to 1 is control over the virus trajectory achieved.

If the time units in the simulation results of Fig. 3 are scaled to match the South Korean dataset, the transitioning period required to achieve control on the spread of the disease corresponds to 5.8 days. It is encouraging to note that such time frame corresponds to actual WHO data.

Investigation of the effects of the rate constant, and the parameters defining the time variation of the step-down constant were explored to achieve the closest correspondence to the modelling results plotted for South Korea and China. The results, tabulated in Table 2, indicate that it is possible to scale the engineering model to reproduce WHO data.

**Table 2.** Growth curve analysis salient for South Korea and China datasets

Country	K growth day^–1^	A/B	C/D	Comments
South Korea	0.6706	6.405–7.205	0.5606–0.5706	
China	0.3364	8.175–8.3009	0.2958	
Model	0.6706	3.9277	0.6078	Scaled to Korean data

The fit of the South Korean data is more promising than the one on the Chinese data, as shown by the data in Table 2.

**Figure 4:**
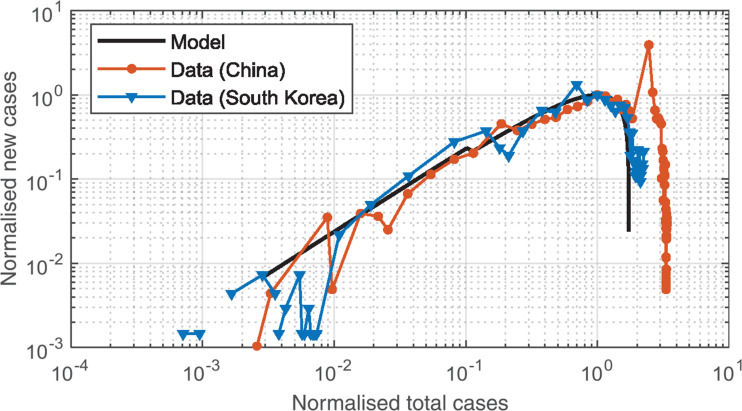
Normalised new cases/unit time versus normalised total cases for South Korean (orange symbols), Chinese (blue symbols) and model (solid black line) data, with normalised new cases axis scaled to emphasise main body of data and good correspondence between South Korean and Chinese results with the model.

While the presentation of the data as shown in Fig. 3 is useful in identifying the departure from the initial exponential growth, the presentation of the data in linear form enables a better comparison of WHO data to the model. For such purposes, the data are normalised to the maximum number of new cases, which also enables the comparison among different datasets. Such a comparison is shown in Fig. 4, where the favourable alignment of the different datasets is evident when one discards the peak in new cases reported in the dataset from China after the first maximum. The spike after the peak in Chinese data is interpreted as an influx of identified cases due to delayed identification.

**Figure 5:**
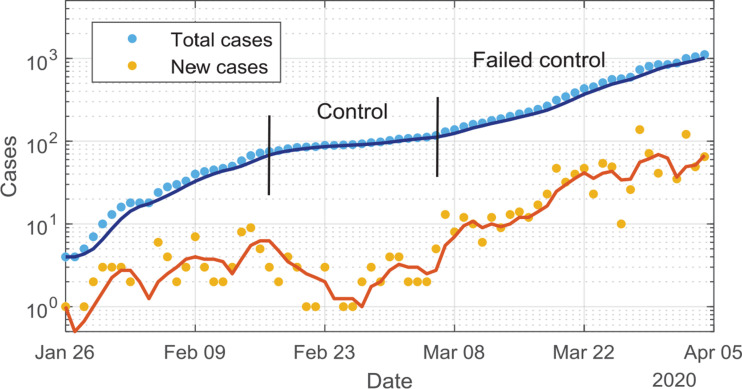
Total cases (blue symbols) and new cases (yellow symbols) per day for Singapore showing evidence of control being first achieved, and then lost. Symbols are WHO data, lines are results from the engineering model.

While in both China and South Korea the analysis of Fig. 4 suggests that the spread of the virus has been limited and the situation seems to be under control, it is possible, based on the results shown in Fig. 2 (orange data), that nations achieve short-term control, but then lose it due to a decline in compliance, or to a premature lift in the isolation/lockdown initiatives. Recent data from Singapore shows evidence of a flattening of the growth rate and new cases per day falling temporarily to zero, before growth is resumed. The WHO data from Singapore are analysed via our engineering model in Fig. 5. Indeed, the increasing slope of the log total cases versus time graph for Singapore after the loss of control shows an accelerating growth rate consistent with a declining K_step-down_, suggesting increasing noncompliance to social distancing and health initiatives.

## Recent developments since the first derivation of the engineering model

Since the presentation of this work in April 2020, during the early part of the COVID-19 pandemic, there have been significant developments. In particular, the summer 2020 months gave the impression that the virus trajectory was for the most part under control across several European countries, until the number of infections started to rise again in many countries, leading to a ‘second wave’ across Europe. The re-examination of COVID-19 data in September 2020 presents the opportunity to examine the outcome of governmental initiatives established in an effort to control the virus trajectory, and to quantify the concerns presented in our original model derivation.

**Figure 6:**
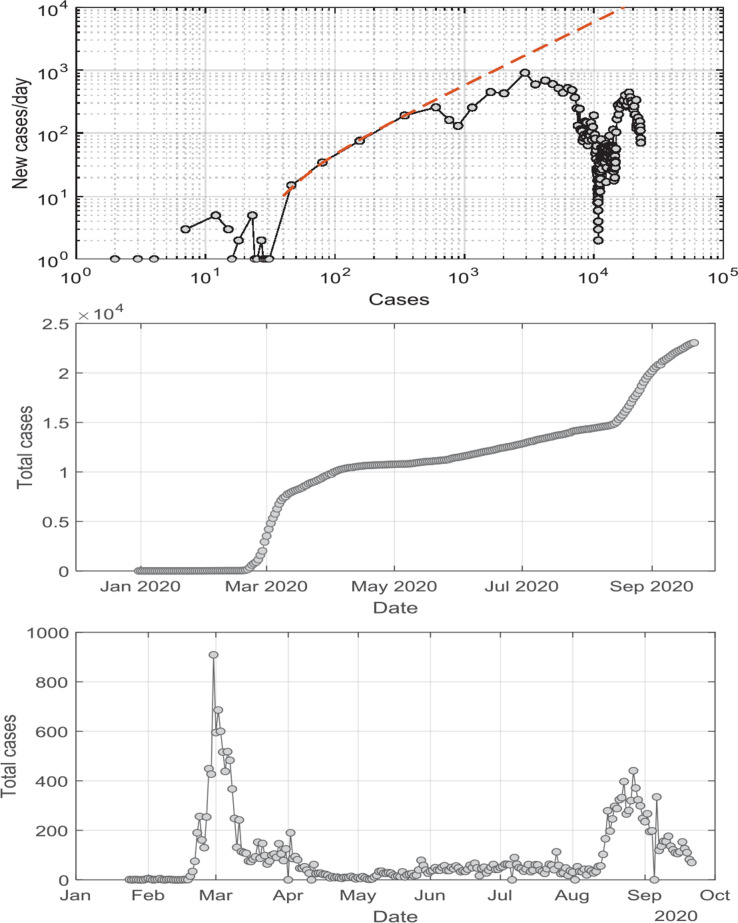
New cases per day versus total cases for South Korea up to 21 August 2020, showing the continued development of the COVID-19 pandemic since the data presented in Fig. 3. The second infection wave, which was managed in summer 2020 is evident in the middle panel, the figure shows total cases versus date, indicating that the initial period of control was being lost; recent progress in managing the viral trajectory is evident as the declining slope of the graph. In the lower panel, the figure shows the new cases per day versus date, which evidences first and second COVID-19 waves.

**Figure 7:**
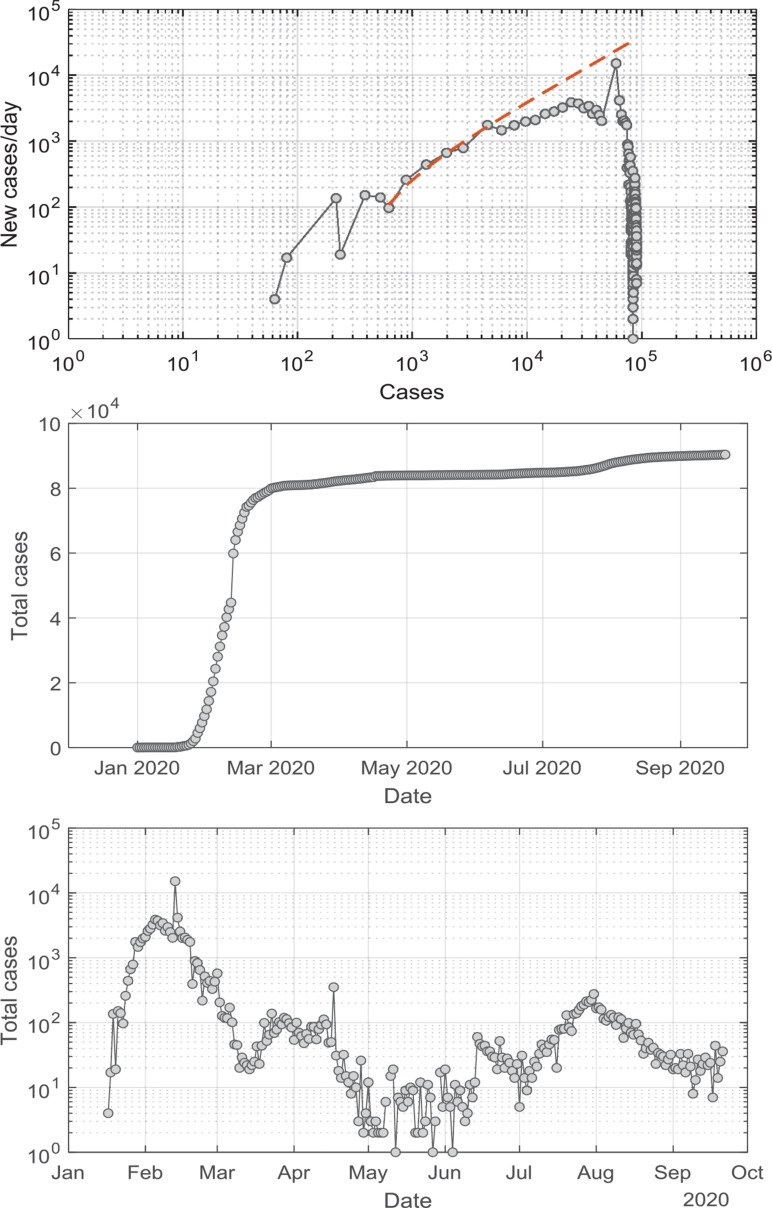
Same as Fig. 6, using WHO data for China up to 21 August 2020. The second infection wave, as well as several subsequent (weaker) infection waves have occurred, but they have all been managed. The lower panel shows at least three subsequent infective waves, of an order of magnitude less severe than the original one. Although all waves have been managed, it is interesting to note the longer recovery times experienced for subsequent infection waves, which would be consistent with lockdown fatigue and less diligent adherence to control directives.

The recent experience of South Korea, whose data are presented in Fig. 6, illustrates that although a second wave was experienced, it resulted in a lower peak cases/day values compared to the first wave, and recent data suggest that the viral trajectory is again being managed successfully in that country, as shown by declining new daily case numbers. The data from China, shown in Fig. 7, show an experience very similar to that just described for South Korea, with second waves being encountered, but managed successfully. One notable feature of the data shown in Fig. 7 is the slower decline in daily cases during the waves of infection subsequent to the first wave. This could be consistent with lockdown fatigue and a reduced degree of adherence to social control measures [39].

Data for COVID-19 infections in the UK up to September 2020 allow us to test whether the engineering model presented here is able to reproduce realistic features such as the control of a first wave via isolation initiatives, and the occurrence of a second wave when such initiatives are relaxed. For this analysis, data explicit for the UK were obtained from the WHO. The data are presented here as new cases per day normalised per million people. The data were acquired from the start date of 26 February 2020, chosen as the date at which new cases per day commenced being consistently greater than zero, and growing. The growth constant k_3_ (see Eq. [8]) was fitted to the initial growth rate of COVID-19 cases during the first wave of viral infection trajectory, prior to lockdown. The parameter k_2_ (see Eq. [2]), was chosen as 0.19 day^–1^, consistent with recent measurements of the infectious periods [40–42].

Significant landmark dates in the COVID-19 trajectory in the UK are: 16 March 2020 – commencement of lockdown (day 20 in our analysis); 20 April 2020 – median date of the peak cases per day plateau, defined as the interval where 5000+ new cases per day was encountered (day 54 in our analysis); 15 July 2020 – commencement of the UK summer school holidays (day 141 in our analysis).

**Figure 8:**
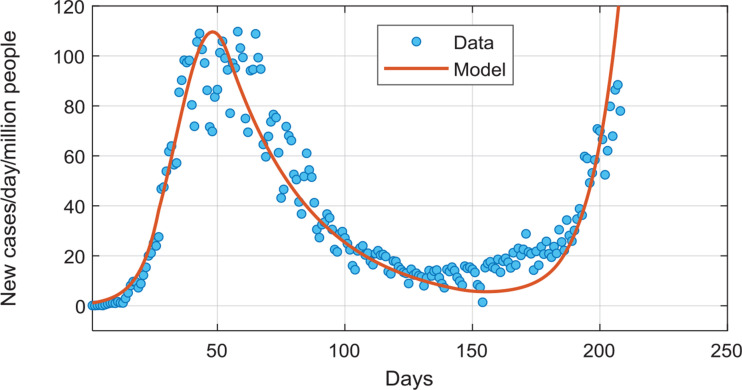
Model predictions (orange line) versus data (blue symbol) for the UK’s first and second infection waves (spring–summer 2020).

Utilising these three dates, we modelled the virus trajectory via applying (e.g., see Eq. [11]) a linear change in the parameter K_step-down_, which is due to the isolation measures, from no restriction measures (K_step-down_ = 0) on day 20 (commencement of lockdown) to a stabilised K_step-down_ = 0.5075 value on day 54 (peak of infections), which represents a relatively good populace adherence to the isolation measures. The latter figure was maintained constant until day 141, where a progressive (linear) return to near-normal behaviour was imposed in the model, reaching K_step-down_ = 0.159 by day 181 of the data series. This date corresponds closely to the end of the UK summer vacation period. As may be seen in Fig. 8, the use of our model, with constants sourced from the literature-reported measurements, and key dates relevant to the propagation of the infection, corresponds favourably to the reported new cases per day over time for the initial infection wave as well as the initial period of the second wave of COVID-19 infections. Examination of the UK data indicates a breakaway value of K_step-down_ = 0.3955–0.4130, the temporary achievement of control being consistent with a figure greater than this, which then relaxed back to a value below this and prompted resumption of increasing new cases/day. As such, the engineering model equipped with known rate constants sourced from observed data is consistent with the right panel of Fig. 2, detailing control gained and lost.

## Discussion of results and conclusions

The engineering model presented here, utilising a linear decline of K_step-down_ over time to reflect increasing compliance to the application of social distancing and lockdown initiatives, achieves good semi-quantitative agreement with the South Korean and Chinese data for the COVID-19 trajectory. A late spike in new cases had been reported in China during the first wave of the pandemic in that region, which is not consistent with the engineering model’s predictions. However, this spike is ascribed to changes in data acquisition and reporting.

The most significant conclusion is that K_step-down_ needs to achieve ∼ 0.92 to mitigate the spread of a virus such as COVID-19; that is to say to the frequency of social interactions needs to be reduced by more than 10-fold compared to the conditions our societies are familiar with. This value is likely to change for regions that are less densely populated than those considered in this analysis.

In the case of South Korea, the time from reaching 100 confirmed cases to the point of maximum case numbers/day (909) was only nine days during the first wave of the COVID-19 trajectory, after which a clear decline in daily case numbers was seen. In China, during the first wave of the COVID-19 trajectory, 18 days were required to reach the daily new cases maximum of 3872 infected Individuals, after which a clear decline is noted. Examination of the model, and of its application to the Singapore case study, indicated that the application of insufficient social intervention will yield the appearance of achieving control, with a reduced number of new cases per day for a period, after which new case numbers will increase and grow. The WHO data related to the first wave of COVID-19 infections in Singapore provides evidence of control achieved and then lost, which is interpreted as consistent with a failure to adhere to restriction measures. Subsequent data show that the viral spread was maintained under relative control.

Examination of the WHO data relative to the first infection wave in the UK with the use of the literature-provided realistic values for the model constants, and changes to the effectiveness of infective transfer as defined by key dates, enabled close modelling of the rise and fall off of infections in the first wave as well as the initial period of the second wave. Of note, the analysis of the engineering model relevant for the UK situation suggests that the viral trajectory can be maintained under control for a relatively modest value of the K_step-down_ constant just above ∼ 0.5. Despite this relatively low level of compliance to the isolation/lockdown initiatives, recent WHO data show that the COVID-19 virus is again spreading quickly throughout the UK. The proximity of the commencement of the second wave of infections to the beginning of the UK summer holiday period is considered as significant in compromising the earlier constraints on infectious transfer achieved during lockdown.

The growth characteristics of the virus trajectory (as defined by the model constants k_1_ and k_2_) are expected to be very sensitive to the environment considered for the study. The rapid growth encountered with the early stages of the COVID-19 pandemic in China, for example, are consistent with a high-density urban population whose behaviours had yet to be modified by personal choice or government directives. As such, compared to the initial social behaviours, high degrees of lockdown were indicated as critical to managing the spread of the disease (which is reflected by high values of K_step-down_). The somewhat slower growth in environments of lower population density and/or where some levels of self-directed social distancing are already in common practice is likely to require lower compliance with isolation strategies (i.e., lower K_step-down_) to achieve control.

Our modelling of the first two waves of the COVID-19 trajectory strengthens the conclusions of our original submission. From the point of view of the goals of this study, it is notable that a relatively simple engineering model is able to capture the trajectory of the virus, using very few parameters that can be easily fitted against the available data.

Analysis of the data and of the model results supports the following conclusions:

- At low levels of infective presence, the number of infected Individuals may be modelled as complying to an exponential approximation, and any departures from this are evidence of changes to the growth rate constants in the propagation of the disease.
- The application of governmental intervention in social distancing and lockdowns can mitigate and control the virus trajectory, but only if a significant decrease in usual social interactions (as defined by high K_step-down_ values in the model) is achieved.
- For insufficient K_step-down_ values, control is not achieved.
- When the compliance with the governmental regulations is sufficiently high, there is a correspondence between the exponential decline in case numbers and the severity of the governmental initiatives. A very much shorter recovery time, lower numbers of infected Individuals, and smaller economic costs are achieved by applying the most severe isolation initiatives, which will need to be applied for a shorter time.
- A reduction in new cases per day does not indicate that control is achieved and can be misleading because a relaxation of compliance to health initiatives will cause a resumption of the exponential growth.
- This simple analysis supports the argument that a severe but short-term lockdown will achieve the fastest reduction in the spread of the virus.

The conclusions just listed were provided on 13 April 2020. Since then, encouraging reports have appeared in the news, suggesting that the lockdown strategies in several countries are yielding the expected positive effects [40]. In some countries such as New Zealand the spread of COVID-19 was under control as early as 28 April (despite subsequent relatively small outbreaks of infections). In response, some governments initiated easing of the isolation/lockdown initiatives during the summer months. Our simple model suggested that it might have been premature to lift isolation/lockdown initiatives.

A review of the viral trajectory conducted based on data retrieved on 21 September 2020 confirmed the development of second infective waves in a number of countries, including all those examined here. Our original conclusions have been vindicated, reinforcing the indication that social interventions robust enough to achieve control are needed, otherwise a less than desirable reduction in R_0_, the basic reproduction number, would be achieved, dragging out the period needed to achieve control, and the associated costs [10]. Consistent with the experience of the initial infection, the data confirming second waves show more severe case numbers in countries where cultural norms valued individual expression over collective security. Our analysis seems to be consistent with the complexity of the situation, as observed by other studies that have emerged in the current literature [4,12,22,23,25]. An informed extrapolation of the implications from our simple engineering model suggests that in order to control the trajectory of the COVID-19 pandemic several subsequent short-to-medium periods of isolation/lockdown initiatives will be inevitable until one or more of the following scenarios occur: (a) a cure has been developed and has become accessible to the population at large; (b) a vaccine has been developed, tested and distributed to large portions of the population; (c) a sufficiently large portion of the population has developed resistance to the COVID-19 virus; or (d) the virus itself has become less aggressive.

It should be recognised that the SIR model presented here is a simple engineering approximation, which does not take into account the physical mechanisms by which COVID-19, or any other virus, spreads. It should also be recognised that the model is used here to fit the available data, which are known to depend on the wide availability of testing. No effort has been made to extrapolate from such available data. The model also does not explicitly quantify the economic nor societal implications of isolation/lockdown initiatives. It instead implicitly assumes a correlation between the number of infected individuals and the negative effects due to COVID-19.

The engineering model presented here does not consider the concept of a critical initial cohort of infected individuals, the potential for growth as defined by the k parameter is the same as for a group of any size. It is appreciated that the k factor is strongly defined by the frequency and nature of contact between individuals and as such population density and the geographical barriers to infection are significant factors in defining the virus trajectory. The model does not consider these influences and considers a numerically and physically static population which has a common k factor.

Similarly, new infective variants had not been encountered at the time of the initial work and as such are not considered herein. It is expected that more infective variants of the virus would lead to an increase in the infection growth rate constant k_1_. All other parameters being constant, such an eventuality would require a higher degree of isolation initiatives to reduce the spread of the virus. As vaccines became available after the first revision of this article was submitted in October 2020, their effect on the virus trajectory has also not been analysed, although it likely can be described as an additional term in the model that describes an increase in the recovered portion of the population.

Areas of possible future investigation would include the effect of connecting several models to investigate the effects of geographical isolation and contact between communities and including the effects of correlation between k factors and different social and demographic groups.

## Data Availability

Data sharing not applicable to this article as no datasets were generated or analysed during the current study.
